# Factors affecting diabetes related hospitalization and in-hospital outcomes of adults with diabetes in south Ethiopia: A prospective observational study

**DOI:** 10.1371/journal.pone.0330735

**Published:** 2025-08-26

**Authors:** Nigist Birhanu, Girma Mamo, Girum Sebsibe shiferaw, Daniel Gizachew, Firehiwot Belayneh, Temesgen Birhanu

**Affiliations:** 1 Dilla University, College of Medicine and Health Sciences, Department of Pharmacy, Dilla, Ethiopia; 2 Jimma University, School of pharmacy, Jimma, Ethiopia; 3 Arbaminch University, Department of Pharmacy, Arbaminch, Ethiopia; Universiti Putra Malaysia, MALAYSIA

## Abstract

**Background and objective:**

Globally, diabetes mellitus is emerging as one of the most common chronic illnesses, and its prevalence has been increasing in the last decades. International Diabetes Federation reports that the number of people with diabetes in Ethiopia is 1.7 million. The prevalence of diabetes mellitus in Ethiopia is rising, and it is expected to increase to 2,031,000 in 2030. Diabetic patients have frequent hospitalization, which is associated with increased morbidity and mortality. The objective of this study was to assess reason for hospitalization and predictors for diabetes related hospitalization and in-hospital treatment outcome of adults with diabetes mellitus in Dilla University, a referral hospital in South Ethiopia.

**Method:**

A hospital-based prospective observational study was conducted at medical, emergency, COVID-19 treatment center, and intensive care unit wards of Dilla University Referral Hospital from September 1, 2020, to May 30, 2021. All adult patients with diabetes mellitus who met the inclusion criteria were consecutively included in the study and followed until discharge or referral or death. To identify the predictors for in-hospital treatment outcome and diabetes related admission, logistic regression was run. Variables with p-value ≤ 0.05 on multivariate regression were considered statistically significant.

**Results:**

A total of 153 patients were enrolled in the study. Diabetic ketoacidosis was the commonest reason for hospitalization (32%), followed by infection (22.2%) and cardiovascular diseases (9.2%). COVID-19 is the major cause of infection-related admission (13.7%). The mean length of hospital stay was 9.92 ± 7.97 days, ranging from 2 to 50 days. Higher proportion were discharged with improvement(71.9%) while 15.7% were self-discharge and 12.24% died in the hospital, mainly due to infections. The presence of comorbidity (AOR = 3.27, 95% CI: 1.39–7.68), obesity (AOR = 3.93, 95% CI: 1.42–10.9), patients with age group of 50–59 (AOR = 0.13, 95% CI: 0.03–0.56), ≥ 60 (AOR = 0.06, 95% CI: 0.018–0.25), and knowledge about diabetic foot ulcer complication (AOR = 0.33, 95% CI: 0.12–0.89) were predictors of diabetes-related admission. Regarding treatment outcome, poor exercise practice (AOR = 2.41, 95% CI: 1.014–5.77, p = 0.046), hypertension (AOR = 3.17, 95% CI: 1.39–7.19, p = 0.006), unsatisfactory knowledge about DM (AOR = 3.5, 95% CI: 1.12–10.9, p = 0.030), and being unmarried (AOR = 3.34, 95% CI: 1.47–7.58, p = 0.004) were predictors of poor treatment outcome.

**Conclusion:**

Generally, this study showed that hyperglycemic emergencies, infections, and cardiovascular diseases were the most common reasons for hospitalization. The mortality rate is high, which is 12.24%, and urges attention to the care of hospitalized patients. Health care professionals should strengthen patient education on glycemic control and self-care practice, since the majority of patients have poor self-care practice and poor metabolic control.

## Background

Diabetes Mellitus (DM) is a metabolic disorder of multiple etiologies, characterized and identified by the presence of hyperglycemia in the absence of treatment, due to defects in insulin secretion, insulin action, or both [1]. According to the International Diabetes Federation (IDF), in 2019 an estimated 463 million adults aged 20–79 years worldwide had diabetes, of which about 79.4% lived in low- and middle-income countries. The prevalence of DM in Africa in 2019, 2030, and 2045 is attributable to 4.7%, 5.1%, and 5.2%, respectively. In 2019, 366,200 deaths (6.8% of all-cause mortality) in the Africa Region were attributable to diabetes [2]. In 2019, IDF reported that the number of people with diabetes in Ethiopia was 1.7 million. The prevalence of DM in Ethiopia is rising, and it is expected to increase to 2,031,000 (3.5%) in 2030 [2,3]. A World Health Organization (WHO) steps survey in Ethiopia found DM prevalence to be 6.5% [4]. Studies in Ethiopia show that there has been an increment in diabetic admissions over time, and the commonest reasons for admission were diabetic complications, infections, and cardiovascular disease (CVD) [5,6].

Diabetes is the third among the five leading global risks for mortality [7]. According to IDF, approximately 4.2 million adults aged 20–79 years died as a result of diabetes and its complications in 2019 [2]. Hospitalization due to diabetes is both an adverse health event and a marker for serious health complications, and is often predictive of disability [8].

In America, the cost of hospitalization due to DM in 2008 was $83 billion [9]. According to IDF, total diabetes-related health expenditure reached 760 billion United states Dollar (USD) in 2019, and the expenditure was also estimated to reach 825 billion USD by 2030 and 845 billion USD by 2045 [2].

Diabetes-related complications often result in patient admissions to hospitals and are also a reason for significant morbidity and mortality among people with diabetes [10]. A study done in Turin, Italy showed that more than 50% of diabetic patients had been admitted to the hospital at least once a year for any cause, and 30% had multiple admissions [11]. A study done in Kuwait showed that DM was the principal or secondary diagnosis in 40.6% of hospitalizations, and patients with diabetes were significantly older and had longer hospital stays compared to non-diabetic patients [12].

In Ethiopia, nearly one-third of diabetic patients face one or another form of acute complications, and approximately half of them face at least one chronic complication which leads to hospitalization [13], and Diabetes ketoacidosis (DKA) is a reason for 11% of hospital admissions [14]. A retrospective study from Addis Ababa showed that the main reason for admission of patients with Type 2 Diabetes Mellitus (T2DM) was diabetic foot ulcer (39%) and CVD (21%), while DKA (62%) is the main reason for admission of patients with Type 1 Diabetes Mellitus (T1DM. A similar study revealed that the overall prevalence of DM hospital admission was found to be 6.5%, with a mortality rate of 21% [15].

As hospitalizations are both costly and can have a significant impact on a patient’s quality of life, identification of risk factors for hospitalization and appropriate risk management plans should be developed to prevent or appropriately manage serious complications associated with diabetes. The reduction of these diabetes-related complications would reduce direct health costs by decreasing the frequency of hospital stays and mortality [16].

To the best of the investigator’s knowledge, although extensive research has been conducted globally on the reasons and predictors for hospitalization and in-hospital treatment outcomes among adults with diabetes mellitus, only limited data exist in Ethiopia. While studies have been conducted in the Southern Nations, Nationalities, and Peoples’ Region (SNNPR), the information available is inconsistent and insufficient to form a comprehensive understanding. Moreover, no study has specifically examined these factors at Dilla University Referral Hospital. Therefore, this study aims to assess reason for hospitalization and predictors for diabetes related hospitalization and in-hospital treatment outcome of adults with diabetes mellitus in Dilla University, a referral hospital in South Ethiopia

### Methods and participants

#### Study setting and period.

This study was conducted from September 1, 2020, to May 30, 2021, at Dilla University Referral Hospital (DURH), SNNPRS, Ethiopia. Dilla Town is the administrative city of the Gedeo Zone, bordered on the east, south, and west by the Oromia Region, and on the north by the Sidama Region. The hospital is located in Dilla Town, 360 km from the capital, Addis Ababa. This research was conducted in the medical, emergency, Coronavirus Disease of 2019 (COVID-19) treatment center, and Intensive Care Unit (ICU) wards of DURH.

#### Study design and participants.

The study design is a hospital based prospective observational study.The source population was all adult patients with diabetes mellitus that were admitted to DURH while all adult diabetic patients admitted to DURH medical, emergency, COVID19 treatment center, and ICU wards during the study period and who fulfill the eligibility criteria were the study population

#### Inclusion and exclusion criteria.

Patients with an age of 15 years and above, patients diagnosed with T1DM, patients diagnosed with T2DM, and patients who were admitted for >24hrs were included for the study while psychiatric patients, incomplete patient data, pregnant women, and patients who were unwilling to give informed consent were excluded.

#### Sample size and sampling technique.

All diabetic patients admitted to the medical, emergency, COVID-19 treatment center, and ICU wards during the data collection period who met the inclusion criteria were consecutively included in the study and followed until discharge, referral to facilities outside DURH, or death. The sample size was determined by using a simple population formula. Using a proportion from a previous study from Jimma University, in which 21 (23.6%) patients had poor treatment outcomes (0.236) [6], Z (standardized normal distribution value at 95% CI) of 1.96, margin of error (d) of 5%, and P (an estimated prevalence rate), the initial sample size calculated was 277 patients. However, the number of diabetes patients admitted to medical, emergency, COVID-19 treatment center, and ICU wards in the previous twelve months was only 225. Hence, the finite population correction formula was applied, and the corrected sample size became 124 patients. Adding 10% for the nonresponse rate, the final sample size became 136 patients.

**Sample size calculation for poor treatment outcome:**


$$n_{i}=\frac{{\left(z_{\alpha /2}\right)}^{2}pq}{d^{2}}$$

$$n_{i}=\frac{\left(1.96{)}^{2}(0.236)(0.764\right)}{(0.05{)}^{2}}=277$$

Where Z = the standard normal value and equals 1.96 at a 95% confidence interval. n_i_ = sample size, P = an estimate prevalence rate,

q=1-p disease free values, d=margin of error (5%)

Since the total number of diabetes mellitus patients is less than 10,000 the following correctional formula is used.


$$n_{f}=\frac{n_{i}*N}{n_{i}+N}$$

$$n_{f}=\frac{277*225}{277+225}=124$$

$$n_{f}=124+10\%\text{ non}-\text{respondent rate}$$

$$n_{f}=\text{136}$$

Where n_i_ = initial sample size which is 277. N = Total expected population within 6month. n_f_ = calculated sample size

**Sample size calculation for reason for hospitalization:**


$$n_{i}=\frac{{\left(z_{\alpha /2}\right)}^{2}pq}{d^{2}}$$

$$n_{i}=\frac{\left(1.96{)}^{2}(0.337)(0.663\right)}{(0.05{)}^{2}}=336$$

Where Z = the standard normal value and equals 1.96 at a 95% confidence interval. ni = sample size, P = an estimate prevalence rate, q = 1-p disease free values, d = margin of error (5%).

Since the total number of diabetes mellitus patients is less than 10,000 the following correctional formula is used.


$$n_{f}=\frac{n_{i}*N}{n_{i}+N}$$

$$n_{f}=\frac{336*225}{336+225}=134$$

$$n_{f}=134+10\%\text{ non}-\text{respondent rate}$$

$$n_{f}=\text{147}$$

Where ni = initial sample size which is 336, N = Total expected population within 12 months, nf = calculated sample size.

### Study variables

#### Independent variables.

Sociodemographic: Age, Sex, Marital status, Residence, Body Mass Index (BMI),Educational status, Occupation, Monthly income, Regular follow up, and family history.

Behavioral measurements: Diabetes Self-care, Knowledge about DM and its complications, Alcohol drinking, and Medication adherence.

Disease Related factors: Type of DM, Duration of DM, Presence of Comorbidities, number of comorbidities, DM complication, previous hospitalization, blood glucose level, type of ward, and length of hospital stay

Medication related factors: Ant diabetics, other medications (for co-morbidities, primary or secondary prevention of Coronary artery disease), and number of Medications.

#### Dependent variable.

- Reasons for hospitalization
- Treatment outcome

**Reason for hospitalization:** any factor that is considered as immediate cause of hospital admission of diabetic patients [6].And classify admission by:

(i) **Diabetes-related admission:** attributed to short term (DKA, Hyperglycemic Hyperosmolar State (HHS), hyperglycemia, or hypoglycemia) or long term complications of diabetes like neurological, renal, diabetic, foot ulcer ophthalmic and cardiovascular complications [6,16]
(ii) **Non-diabetes-related hospitalizations:** included all other reasons for hospitalization not classified as diabetes-related [16].

**Treatment outcome:** is the condition of the patient at the end of the hospital stay.

**Good treatment outcome:** admitted patient discharged with the improvement which is decided by the health care team

**Poor treatment outcome:** in-hospital death, self-discharge and referral was considered as poor treatment outcome [6].

**Data collection methods and tools:** Relevant information was collected from patient charts [current diagnosis, previous history of admission, comorbidities, diabetic complications, blood glucose, medication given, and laboratory results] using a checklist. Information about patient sociodemographics, disease duration, knowledge about DM complications, medication adherence, self-care activities, and family history of diabetes was obtained by interviewing the patient using a structured questionnaire from previously done literature [6,17]. A standard questionnaire tool was used [18–20]. The data collection tool was first developed in English and translated to Amharic and Gediofa, then back-translated to English to check its consistency before data collection.

Assessment of self-care activities has five domains (general diet, specific diet, exercise, foot care, and blood glucose testing). For all domains, frequency of self-care activity in the last seven days was measured. For each domain, the mean was calculated and categorized as good for scores above the mean value and poor for scores below the mean value. The overall mean score was calculated by summation of the mean score for general diet, specific diet, exercise, foot care, and blood glucose testing divided by the sum of the number of questions under each scale [20,21]. After calculating the mean score, patients were categorized as good general diet plan if scored ≥ 2.9, good specific diet plan if scored ≥ 3.5, good foot care if scored ≥ 2.5, good exercise if scored ≥ 2.8, and good self-blood glucose testing if scored ≥ 2.7. After calculating the overall mean score, it was classified as having good self-care practice if the patient scored ≥ 2.9 or poor self-care practice if the patient scored < 2.9. To assess knowledge of diabetes complication and diabetes knowledge, the 24-item version of the Diabetes Knowledge Questionnaire (DKQ-24) was employed [18]. If study participants answered greater than 80% of DKQ-24 items, it was considered that they had good knowledge about DM [22].

### Data collectors

One hospital pharmacist and three nurses were recruited as data collectors, and a one-day training was provided to them on interview techniques and assessment of anthropometric data, including height and weight, using a measuring tape and a portable digital scale..

#### Data analysis and interpretation.

The data was entered into EpiData version 3.1 and exported to Statistical Package for the Social Sciences (SPSS) version 26 for statistical analysis. Descriptive statistics like frequency, proportion, median, mean, and standard deviation were employed to describe the sociodemographic, clinical, and behavioral characteristics of patients. To identify the predictors, bivariable and multivariable logistic regression was run, and the results of the bivariable and multivariable logistic regression analysis were reported as crude and adjusted odds ratios at 95% confidence intervals (95% CI). Variables with a p-value < 0.25 on bivariable logistic regression were recruited for multivariable logistic regression. In multivariable logistic regression, backward conditional analysis was used, and those variables with p-value ≤ 0.05 were considered statistically significant and predictors of diabetes-related hospital admission and poor treatment outcome. Hosmer–Lemeshow goodness-of-fit test was done to check model fitness.

### Data quality assurance

Before the actual data collection, a pretest was conducted using the data collection tool on eight patients (5% of 147) at DURH, selected randomly from DM patients.The pretested patients were not included in the study, and appropriate adjustments were done to the data collection tool. Training was given to data collectors about the objective, relevance, appropriate use of the data collection tool, focusing on the uniform interpretation of questions, strict use of study criteria, obtaining written and informed consent from study patients, confidentiality of the data to be collected, and respondent’s rights. In addition to this, the principal investigator was supervising the data collectors during data collection through phone calls. The collected data were checked for completeness and consistency daily.

### Ethical consideration

This study was conducted after a letter of ethical clearance was obtained from the Institutional Review Board (IRB) of the Institute of Health, Jimma University. Official permission was obtained from Dilla University Referral Hospital’s clinical director before data collection. In addition to this, informed written consent was obtained from each study participant concerning their willingness to take part in the study after explaining the objective of the study. Privacy and confidentiality were ensured during the review of patients’ charts by data collectors. Thus, the name and addresses of patients were not included in the data abstraction format.

### Operational definitions and definition of terms

**Adequate knowledge about DM complications:** The knowledge regarding the complications of diabetes was considered adequate when a patient could mention ≥3/5 complications mentioned in the data collection tool.

**Some knowledge about DM complications:** The knowledge regarding the complications of diabetes was considered some when a patient could mention 1–2/5 complications mentioned in the data collection tool.

**No knowledge about DM complications:** The knowledge regarding the complications of diabetes was considered no knowledge when a patient could not mention at least one of the five major DM complications mentioned in the data collection instrument [17,23].

**Knowledge about DM:** the response variable was determined from the DKQ-24, with three response options for each question: yes, no, don’t know. One mark was given for right answer and zero for wrong answer or doesn’t know. Patients were classified as having **poor knowledge** (<60%), **Acceptable knowledge** (60–80%) and **good knowledge** (>80%) [22].

**Ever use alcohol:** was defined as respondents who admitted to having ever used alcohol.

**Current use alcohol:** was defined as the proportion who took alcoholic drinks within the last 30 days.

## Results

### Overview of the study

**Fig 1:**
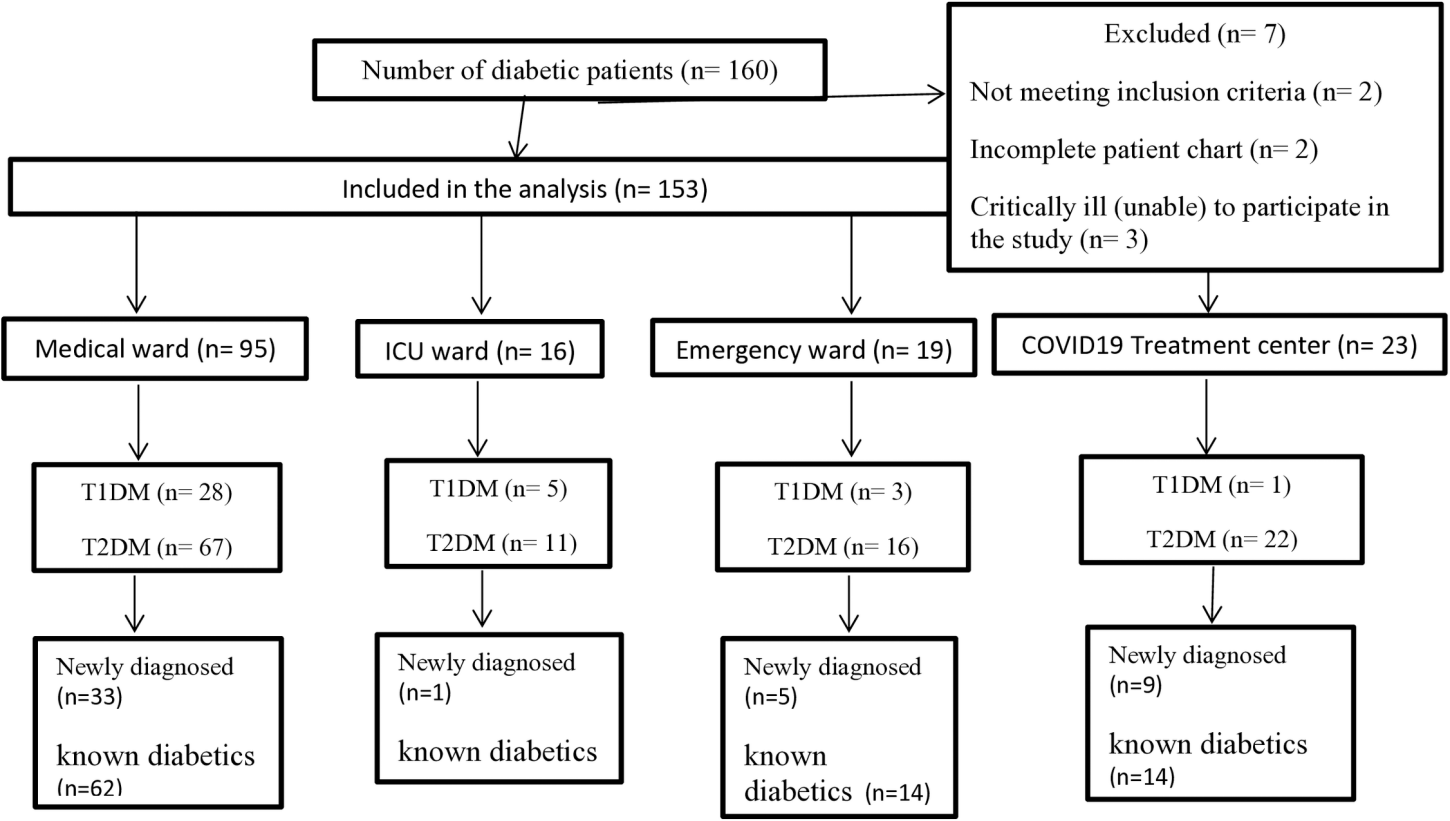
Flow chart of enrollment of adults with diabetes mellitus admitted to DURH medical, emergency ICU, and COVID-19 treatment center wards, September 1 to May 30, 2020/2021.

During the study period, a total of 160 diabetic patients were admitted to medical, ICU, emergency ward, and COVID-19 treatment center, of which 153 were included in the analysis (Fig 1).

### Socio-demographic characteristics of participants

More than half, 87(56.9%) of patients were males. The mean age of the participants was 48.4 ± 19.2 years with a range of 15–105 years; one-third of the patients were older than 60years 51(33.3%). Eighty-nine (58.2%) of them were from urban and forty-eight (31.4%) were unable to read and write. Eighty (52.3%) of the patients do not have a family history of diabetes (Table 1).

**Table 1:** Socio-demographic characteristics of adults with diabetes mellitus admitted to DURH medical, emergency ICU, and COVID- 19 treatment center wards, September 1 to May 30, 2020/2021.

Variables	Category	Frequency N = 153(%)
Sex	Male	87(56.9)
	Female	66(43.1)
Age	15–29	29(19)
	30–39	22(14.4)
	40–49	27(17.6)
	50–59	24(15.7)
	>=60	51(33.3)
Residence	Urban	89(58.2)
	Rural	64(41.8)
Occupation	Government employee Merchant	28(18.3) 39(25.5)
	Student	18(11.8)
	Farmer	23(15)
	Housewife	24(15.7)
	Retired	21(13.7)
Marital status	Married	97(63.4)
	Unmarried	56(36.6)
Family history of DM	Yes	73(47.7)
	No	80(52.3)
Average monthly income in Birr	<=500	30(19.6)
	501–1500	46(30.1)
	1501–2500	18(11.8)
	2501–3500	19(12.4)
	>=3500	40(26.1)
Ever Alcohol Drinking	Yes	54(35.3)
	No	99(64.7)
Current use alcohol(n = 54)	Yes	49(32)
	No	5(3.3)
Educational states	Unable read wright	48(31.4)
	read & wright only Primary	45(29.4) 9(5.9)
	Secondary	17(11.1)
	College and above	34(22.2)
Smoking	Yes	22(14.4%)
	No	131(85.6)
BMI	Normal weight	56(36.6)
	Overweight	47(30.7)
	Obese	50(32.7)

### Clinical characteristics

#### Disease related factors.

Out of 153 patients, 105 (68.6%) were known diabetics, while 48 (31.4%) were newly diagnosed. The mean duration of diabetes for known diabetics was 5.8 ± 4.7 years; the majority of them were diagnosed in the last 5–10 years. Seventy-six patients out of 105 known diabetics had regular follow-up. Seventy-five patients had a prior history of admission. More than half of the patients, 77 (50.3%), had chronic comorbidity. Among the total patients, 38 (24.8%) had long-term diabetic complications, and neuropathy (12 [31.6%]) was the most common type of complication (Table 2).

**Table 2:** Disease related factors among adults with diabetes mellitus admitted to DURH medical, emergency ICU, and COVID-19 treatment center wards, September 1 to May 30, 2020/2021.

Variables	Category	Frequency (%)
Long-term Diabetic Complications	Present	38(24.8)
	Absent	115(75.2)
Specific Diabetic Complications (N = 38)	Neuropathy	12(31.6)
	Foot ulcer	10(26.3)
	Nephropathy	7(18.4)
	Retinopathy	4(10.5)
	Foot ulcer, neuropathy	5(13.2)
Number of complications	1-2 complications	32(84.2)
	≥ 3 complications	6(15.8)
Co-morbidities	Present	77(50.3)
	Absent	76(49.7)
Specific Co-morbidities (N = 153)	Hypertension	52(34)
	Obesity	32(20.9)
	Coronary Artery Disease	25(16.3)
	Congestive Heart Failure	17(11.1)
	Chronic Kidney Disease	12(7.8)
	Stroke	3(2)
	Other*	7(4.6)
Number of Co-morbidities	1-2 Co-morbidities	58(75.3)
	≥ 3 Co-morbidities	19(24.7)
Type of diabetes mellitus	T1DM	37(24.2)
	T2 DM	116(75.8)
Duration of living with DM	newly diagnosed DM	48(31.4)
	Known DM	105(68.6)
	<1 year	20(19)
	1–5 year	27(25.7)
	5–10 year	36(34.3)
	10–15 year	14(13.3)
	≥15 year	8(7.6)
Prior history of hospitalizations	No	78(51)
	yes	75(49)
	1 time	19(25.3)
	2 times	24(32)
	3 times	14(18.7)
	4 times	18(24)
Regular follow up	Yes	76(72.4)
	No	29(27.6)

*Human Immunodeficiency Virus (HIV), Asthma, Chronic Liver Disease (CLD), Epilepsy.

### Medication related factors

Of 105 known diabetic patients, before admission, 45 (42.9%) patients were on insulin, 34 (32.4%) patients were on metformin and glibenclamide, 16 (15%) patients were on metformin, and 10 (9.5%) patients were on a metformin and insulin combination. Before admission, 44 patients were taking non-antidiabetic medications, from which 13 (29.5%) patients were on amlodipine, 10 (22.7%) patients were on diuretics, 9 (20.5%) patients were taking statin and aspirin, 9 (20.5%) patients were on statins and enalapril, and 3 (4.5%) patients were on other medications such as HAART, phenytoin, and bronchodilator. Thirty-five patients had discontinued their medication (Table 3).

**Table 3:** History of medication use before admission among adults with diabetes mellitus admitted to DURH medical, emergency, ICU, and COVID- 19 treatment center wards, September 1 to May 30, 2020/2021.

Types of medications	Frequency (%)
Ant diabetic Medications (N = 105)	
Insulin	45(42.9)
Metformin and glibenclamide	34(32.4)
Metformin	16(15)
Metformin and insulin	10(9.5)
Cardiovascular drugs (N = 44)	
Amlodipine	13(29.5)
Diuretics	10(22.7)
Statin and Aspirin	9(20.5)
Statins & enalapril	9(20.5)
Other*	3(4.5)
Discontinue any medication before admission(N = 153)	
Yes	35(22.9)
No	118(77.1)

*HAART & bronchodilator.

After admission in all 153(100%) patients insulin was used to manage hyperglycemia while metformin is used in 31(20.26%) patients in addition to insulin. In addition to anti-diabetics, several medications were used in the management of hospitalized diabetic patients. Among those, anti-infective111 (72.5%) followed by cardiovascular drugs58 (37.9%) were the commonest prescribed medications (Table 4).

**Table 4:** Patterns of medication use among adults with diabetes mellitus admitted to DURH medical, emergency, ICU, and COVID- 19 treatment center wards, September 1 to May 30, 2020/2021.

Types of medications	Frequency, N = 153(%)
Ant diabetic Medications	
Insulin	153(100)
Metformin	31(20.26)
Anti-infective	111(72.5)
Cephalosporin	104(68)
Azithromycin	41(26.8)
Glucocorticoid	27(17.6)
Vancomycin	26(17)
Metronidazole	23(15)
Anti TB	12(7.8)
Fluoroquinolone	4(2.6)
HAART	2(1.3)
Cardiovascular drugs	58(37.9)
Anti-lipidemic agents	42(27.5)
Diuretics	40(26.1)
ACEIs	29(19)
Beta blockers	24(15.7)
Drugs affecting GIT	49(32)
Antiulcer	57(37.3)
Antiemetic	26(27)
Laxatives	12(7.8)
Drugs affecting blood	49(32)
Analgesics	47(30.7)
Other*	8(5.2)

*Artesunate, hydrocortisone, phenytoin, Amitriptyline, Coartem®, KCL.

### Self-care behavior adherence

As reported by the respondents, 70 (45.8%) patients had good adherence, and 83 (54.2%) patients had poor adherence to their self-care behaviors. Regarding the diabetes self-care practice domains, the majority of the patients had poor diabetes self-care practice across all domains: 77 (50.3%) for general diet, 108 (70.6%) for specific diet, 88 (57.5%) for exercise, and 83 (54.2%) for recommended blood sugar testing. However, more than half of the patients, 81 (52.9%), had good foot care practice (Table 5 and Table 6).

**Table 5:** The mean score of individual and overall self-care behaviors of adults with diab/etes mellitus admitted to DURH medical, emergency ICU, and COVID- 19 treatment center wards, September 1 to May 30, 2020/2021.

Variable	Item	mean score *	(±SD)	Mean of Domain	overall mean**
General Diet	-Number of days followed a healthful eating plan	2.92	1.37	2.9	2.9
	-average, over past month, number of days followed eating plan	2.92	1.42		
Specific Diet	Number of days you eat five or more fruits & vegetables	2.79	1.52	3.5	
	Number of days you eat high fat foods	4.21	1.7		
Exercise	How many days; participate at least 30 minutes of activity last 7 days?	2.73	2.4	2.5	
	How many days, participate in specific exercise session last 7 days?	2.28	2.52		
Blood Sugar Testing	How many of last 7days you test your blood sugar?	2.77	2.54	2.8	
	How many of last 7days test blood sugar, recommended by your health care provider?	2.79	2.61		
Foot care	How many of last7 days you check your feet?	2.63	2.47	2.7	
	How many of last7 days you inspect inside of your shoes?	2.67	2.41		

*Mean score of individual self-care = sum of the number of days self-care practice/ Total number of patients (153).

**Overall mean score of self-care = the sum of each means/10.

**Table 6:** Distribution of diabetes self-care practice domains of a patient admitted to DURH medical, emergency ICU, and treatment center wards, September 1 to May 30, 2020/2021.

Self-care practice domains	good practice	poor practice
General Diet	76(49.7%)	77(50.3)
Specific Diet	45(29.4%)	108(70.6)
Exercise	65(42.5%)	88(57.5)
Blood Sugar Testing	70(45.8%)	83(54.2)
Foot care	81(52.9%)	72(47.1)
Overall self-care practice	70(45.8%)	83(54.2)

### Diabetes Knowledge

Diabetes knowledge was poor in 118 (77.1%), acceptable in 33(21.6%), and good in 2(1.3%) patients (Table 7).

**Table 7:** Knowledge about diabetes mellitus among adults with diabetes mellitus admitted to DURH medical, emergency ICU, and COVID- 19 treatment center wards, September 1 to May 30, 2020/2021.

Variables	Frequency, N = 153(%)
Knowledge	
Poor knowledge (<60%)	118(77.1)*
Acceptable knowledge (60–80%)	33(21.6) **
Good knowledge (>80%)	2(1.3) **

*118 has Unsatisfactory knowledge (<60%), **35 has Satisfactory knowledge (>60%).

### Knowledge about diabetes complications

Of a total of 153 participants 80 (52.3%) had no knowledge,64 (41.8%) had some knowledge and 9(5.9%) had adequate knowledge about diabetic complications. Of this, knowledge neuropathy accounted for the highest rank, 56 (36.3%) of the given complications (Table 8).

**Table 8:** Knowledge about diabetic complications among adults with diabetes mellitus admitted to DURH medical, emergency ICU, and COVID- 19 treatment center wards, September 1 to May 30, 2020/2021.

Variables	Frequency, N = 153(%)
Category of Knowledge about Diabetic Complications	
No knowledge	80(52.3)
Some Knowledge	64(41.8)
Adequate Knowledge	9(5.9)
Knowledge about specific DM complication	
Neuropathy	56(36.6)
Foot ulcer	32(20.9)
Kidney complication	23(15)
Retinopathy	15(9.8)
Cardiovascular disease	4(2.6)

### Reasons for hospitalization

Of a total of 153 patients, 95(62.1%) and 58(37.9%) patients were admitted because of diabetes and non-diabetes related cases respectively. DKA was the most common reason for hospitalization attributed to admission of 49 (32%) patients followed by infection 34 (22.2%). Among infectious cases, COVID-19 was the commonest one, 21(13.7%). The mean admission blood glucose was 344.37 ± 151.3 mg/dl which ranges from 26 to 600 mg/dl (Table 9).

**Table 9:** Reasons for hospitalization and admission blood glucose level of patients admitted to DURH medical, emergency, ICU, and COVID- 19 treatment center wards, September 1 to May 30, 2020/2021.

Variables	Category	Frequency, N = 153(%)
Reason for hospitalization	Diabetic related	95(62.1)
	Non-Diabetes related	58(37.9)
Specific Diabetic related	Diabetic ketoacidosis	49(32)
	Hyperglycemia	19(12.4)
	Hyperglycemic hyperosmolar state	18(11.8)
	Diabetic foot ulcer	8(5.2)
	Hypoglycemia	1(0.7)
Non-Diabetes related	Infections	34(22.2)
	COVID19	21(13.7).
	Severe community-acquired pneumonia	7(4.6)
	Tuberculosis bacillus	6 (3.9)
	Cardiovascular Disease	14(9.2)
	Acute coronary syndrome	6(3.9)
	Congestive heart failure	5(3.3)
	Hypertension	3(2)
	Chronic liver Disease	5(3.3)
	Other*	5(3.3)
Admission blood glucose	<=70 mg/dl	4(2.6)
	71-180 mg/dl	23(15)
	181-250 mg/dl	23(15)
	251-350 mg/dl	30(19.6)
	351-450 mg/dl	34(22.2)
	451-599 mg/dl	31(20.3)
	>=600 mg/dl	8(5.2)

*Acute kidney injury, urinary tract infection, GERD.

### Treatment outcome and length of hospital stay

Out of 153 patients who participated in this study, 110 (71.9%), 24 (15.7%), and 19 (12.4%) patients were improved, self-discharged, and died, respectively. This indicated that 43 (28.1%) patients had poor treatment outcomes (self-discharged and died). Of the 19 patients who died, 9 were admitted due to COVID-19, 4 due to DKA, 2 due to HHS, and 1 each due to Acute Coronary Syndrome (ACS), Hypertension (HTN), CLD, and pneumonia admission. Ten patients died within 5 days of admission, 4 within 5–10 days, while the remaining 5 after 10 days of hospitalization. The mean length of hospital stay was 9.92 ± 7.97 days, ranging from 2 to 50 days. Over one-third of the patients, 59 (38.6%), stayed 5–10 days in the hospital (Table 10).

**Table 10:** Treatment outcome and length of hospital stay of adults with diabetes mellitus admitted to DURH medical, emergency, ICU, and COVID- 19 treatment center wards, September 1 to May 30, 2020/2021.

Variables	Category	Frequency, N = 153(%)
**Treatment outcome**	Improved	110(71.9)*
	Self-Discharge	24(15.7)**
	Died	19(12.4)**
**Length of Hospital Stay**	<5 day	49(32)
	5-10 day	59(38.6)
	>10 day	45(29.4)

*good treatment outcome, **poor treatment outcome.

**Fig 2:**
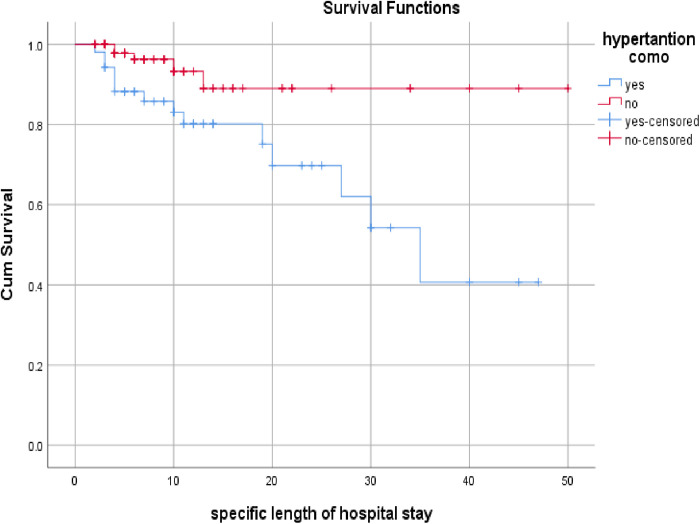
Kaplan-Meir survival curve of diabetic patients admitted to DURH medical, emergency, ICU, and COVID-19 treatment center wards, September 1 to May 30, 2020/2021.

The overall mean time to death after hospitalization was 38.1 ± 2.6 days since from the date of admission. The mean time to death after hospitalization for those who have hypertension is 31.47 ± 3.16 days and 47.5 ± 2.1 days for those who do not have hypertension. The probability of survival for those who have hypertension decrease with time as compared to patients who do not have hypertension with **Log rank test p = 0.009** (Fig 2).

### Predictors of diabetes related hospitalization and treatment outcome

#### Predictors of diabetes related hospitalization.

Although several variables were associated with diabetes-related hospitalization on binary logistic regression analysis, presence of comorbidity, obesity, age groups 50–59 and ≥60, and knowledge about foot ulcer complications remained independent predictors of hospitalization on multivariable analysis.

Accordingly, patients who have comorbidity are 3.27 times more likely to have diabetes-related hospitalization than patients who do not have comorbidity (AOR = 3.27, 95% CI: 1.39–7.68, p = 0.006). Likewise, patients who have obesity (AOR = 3.93, 95% CI: 1.42–10.9, p = 0.008) were more likely to have diabetes-related admission than patients who are not obese.

On the other hand, patients aged 50–59 (AOR = 0.13, 95% CI: 0.03–0.56, p = 0.006), aged ≥60 (AOR = 0.06, 95% CI: 0.018–0.25, p = 0.000), and patients who have knowledge about diabetic foot ulcer complications (AOR = 0.33, 95% CI: 0.12–0.89, p = 0.029) were less likely to experience diabetes-related admission than their counterparts (Table 11).

**Table 11:** Predictors of diabetic related admission of adults with diabetes mellitus admitted to DURH medical, emergency, ICU, and COVID-19 treatment center wards, September 1 to May 30, 2020/2021.

Variables	Diabetes-related admission		Binary logistic regression		Multivariable logistic regression	
NO	YES	COR(95%CI)	P	AOR(95%CI)	P
Comorbidities						
Present	21	56	2.5(1.29-4.96)*	0.003	3.27(1.39-7.68)*	0.006
Absent	37	39	1		1	
Knowledge of DM complications						
No Knowledge	24	56	4.6(1.07-20.2)*	0.039	3.46(0.49-24.3)	0.213
Some Knowledge	28	36	2.5(0.5-11.1)	0.2	4.12(0.6-27.5)	0.143
Adequate Knowledge	6	3	1		1	
Age						
15-29	4	25	1		1	
30-39	2	20	1.6(0.26-9.6)	0.6	1.9(0.29-12.7)	0.493
40-49	8	19	0.38(0.09-1.4)	0.15	0.33(0.07-1.39)	0.131
50-59	13	11	0.13(0.03-0.5)*	0.003	0.13(0.03-0.56)*	0.006
>=60	31	20	0.1(0.03-0.3)*	0	0.06(0.018-0.25)*	0
BMI						
Normal weight	26	30	1		1	
Overweight	20	27	1.17(0.5-525)	0.694	1.09(0.42-2.84)	0.845
Obese	12	38	2.74(1.19-6.32)*	0.018	3.93(1.42-10.9)*	0.008
Type of diabetes						
T1DM	6	31	4.19(1.6-10.8)*	0.003	1.33(0.26-6.65)	0.724
T2DM	52	64	1		1	
Knowledge about Foot ulcer						
Yes	18	14	0.38(0.17-0.8)*	0.018	0.33(0.12-0.89)*	0.029
No	40	81	1		1	
Presence of CAD						
Yes	14	11	0.4(0.17-0.98)*	0.045	0.98(0.31-3.12)	0.98
No	44	84	1		1	
Occupation						
Government employee	8	20	3.3(1.01-10.9)*	0.04	1.39(0.25-7.5)	0.702
Merchant	15	24	2.1(0.7-6.2)	0.16	1.03(0.19-5.43)	0.97
Student	1	17	22.6(2.5-203)*	0.005	3.64(0.19-69.6)	0.389
Farmer	13	10	1.02(0.3-3.3)	0.96	0.43(0.09-1.96)	0.279
Housewife	9	15	2.2(0.6-7.3)	0.19	2.53(0.48-13.4)	0.273
Retired	12	9	1		1	

#### Predictors of treatment outcome.

On multivariable analysis, poor exercise practice, hypertension comorbidity unsatisfactory knowledge about DM, and unmarried remained independent predictors of poor treatment outcomes.

Accordingly, patients who have poor exercise practice are 2.41 times more likely to have poor treatment outcomes than patients who have good exercise practice (AOR = 2.41, 95%CI:1.014–5.77, p = 0.046). Similarly, patients who have hypertension and unsatisfactory knowledge about DM have 3.17 and 3.5 times more likely to have poor treatment outcomes than their counterparts (AOR = 3.17, 95%CI: 1.39–7.19, p = 0.006, AOR = 3.5, 95%CI: 1.12–10.9, p = 0.030) respectively. In addition, unmarried participants were 3.34 times more likely to have poor treatment outcomes (AOR = 3.34, 95%CI: 1.47–7.58, p = 0.004) (Table 12).

**Table 12:** Predictors of treatment outcome of diabetic patients admitted to DURH medical, emergency, ICU, and COVID- 19 treatment center wards, September 1 to May 30, 2020/2021.

Variables	Treatment outcome		Univariate Analysis		Multivariable logestic regression	
	Good	poor	COR (CI 95%)	P	AOR (CI 95%)	P
Mean of exercise practice						
Poor (<2.5)	56	32	2.8(1.28-6.1) *	0.01	2.4(1.014-5.7)*	0.046
Good (>=2.5)	54	11	1		1	
Knowledge about DM						
Unsatisfactory (<=60%)	80	38	2.85(1.025-7.9)	0.045	3.5(1.12-10.9)*	0.030
satisfactory(>60	30	5	1		1	
Residence						
Urban	70	19	0.45(0.2-0.9)*	0.03	0.5(0.22-1.11)	0.090
Rural	40	24	1		1	
Hypertension comorbid						
Present	30	22	2.79(1.34-5.8)*	0.006	3.17(1.39-7.19)*	0.006
Absent	80	21	1		1	
Discontinue medication before admission						
Yes	20	15	2.4 (1.09-5.3)*	0.03	1.33(0.5-3.5)	0.564
No	90	28	1		1	
Marital status						
Married	80	17	1		1	
unmarried	30	26	4.07(1.94-8.56)*	0.000	3.34(1.47-7.58)	0.004
Age						
15-29	26	3	0.17(0.04-0.67)*	0.01	0.25(0.05-1.15)	0.077
30-39	15	7	1.38(0.25-2.08)	0.54	1.16(0.31-4.27)	0.815
40-49	21	6	2.25(0.15-1.28)	0.13	0.75(0.21-2.62)	0.653
50-59	17	7	1.56(0.2-1.8)	0.39	0.94(0.28-3.18)	0.928
>=60	31	20	1.00	1.00	1.00	1.00

## Discussion

In this study, the most common reasons for hospitalization were hyperglycemic emergencies, infections, and CVDs. While most patients were discharged with improvement, a significant number died during hospitalization. Hyperglycemic emergencies (DKA and HHS) accounted for 43.8% of admissions—higher than reported in Saudi Arabia (21%), Uyo Nigeria (18.7%), and Harar (24.6%) [24–26]. This may reflect poor glycemic control in the study setting, possibly due to a higher proportion of newly diagnosed DM patients presenting with acute complications. Low self-care practices and the impact of the COVID-19 outbreak may also have contributed to uncontrolled hyperglycemia. However, the rate was lower compared to studies from Nigeria (57.8%) and Uganda (48.3%) [9,27], which could be due to differences in sample size, study design, and setting.

The overall diabetes-related admission rate was 62.1%, which is comparable to the result from Nigeria (62.7%) [28]. But higher than reports from Jimma (48.3%), Barbados (33.6%), Addis Ababa (54%), England (50.8%), and Harer (30.6%) [6,15,16,26,29]. This difference may be due to poor metabolic control, limited knowledge about diabetes and its complications, and inadequate self-care practices at DURH. In this study, 32% of diabetes-related admissions were due to DKA, which is similar to findings from Jimma (33.7%) [6]. But higher than studies in Nigeria, Barbados, and Addis Ababa, where diabetic foot ulcers were the leading causes of admission (37%, 89%, and 31%, respectively). However, diabetic foot ulcers accounted for only 5.2% of diabetes-related hospital admissions in this study [15,28,29].

Infection was the second leading cause of admissions (22.2%), from which 13.7% of admissions were due to COVID-19, and this is comparable with the study done in Kuwait where 22.8% of diabetic patients were admitted with infection [12], but it is higher than studies from Jimma, Saudi Arabia, Addis Ababa, and Harar where infections accounted for 19%, 16%, 13%, and 9.7% of diabetes patients’ hospital admissions, respectively [6,15,24,26]. The difference might be due to the current pandemic of COVID-19. Also, uncontrolled diabetes (56.2%), which is high in our setup, might negatively affect the functions of cells in the immune system and contribute to infection development. However, it was lower compared with a study done in Nepal and Uganda where infections were seen in 84.1% and 27.7% of patients, respectively [27,30]. The difference may be due to the majority of patients in Uganda presenting with long-term complications (56.7%) compared to ours (24.8%), which may predispose patients to infections. Apart from hyperglycemia, chronic complications of diabetes may predispose patients to infections [31].

Cardiovascular disorders, the third cause of admission, accounted for 9.2%. It is lower than the study done in Kuwait (53.6%) [12]. This might be due to differences in socioeconomic status among the population, which could affect the prevalence of CVDs. It is lower compared to the study done in Jimma, where 18.0% of diabetes hospital admissions were due to CVD [6]. This could be due to differences in the duration of diabetes among patients, since the duration of diabetes has been a risk factor for an increased risk of CVD [32]. In the study conducted in Jimma, about 12.4% of patients have been living with diabetes for ≥15 years, while only 7.6% of patients involved in this study have been living with diabetes for the same length of time.

Out of 153 patients, 110 (71.9%) were discharged home with improvement, which is comparable with 74.6% and 72% reported from Nigeria and Addis Ababa, respectively [15,28], but lower than 76.4%, 89.4%, and 85.8% reported from Jimma and two studies from Nigeria, respectively [6,9,25]. This may be because the number of patients who were self-discharged was higher in DURH than in these settings. The rate of self-discharge was 15.7%, which is higher than the study from Jimma and Nigeria, which reported 6.74%, 2.4%, and 13.3% [6,9,25]. The variation may be related to differences in the socioeconomic status of the patients, since those with lower economic status are unable to stay in hospital for a longer period.

In our study hospital, mortality was 12.1%, which was comparable with 10.6%, 10.8%, 13%, 11%, and 11.2% reported from studies done in Addis Ababa, Jimma, Saudi Arabia, Nigeria, and Uganda, respectively [5,6,24,27,33]. But the mortality rate was higher compared with results from three studies from Nigeria, Japan, and Harer, Ethiopia: 3.4%, 8.1%, 0.8%, 3.1%, and 4.4%, respectively [9,25,26,28,34]. These differences may be attributed to institutional differences in the care of hospitalized diabetic patients, admission ward differences, severity of disease, and variation in reason for admission. In our study, infection was the second leading cause of admission, from which COVID-19 was the leading one. This might be the reason for the majority of deaths. There is an increment of risk of severity and mortality associated with COVID-19 in patients with DM [35].

Comorbidity, obesity, age groups 50–59 and ≥60, and knowledge about foot ulcer complications were predictors of diabetes-related hospital admission on multivariable analysis.

Patients who knew about diabetic foot ulcer complications were less likely to have diabetes-related admission than those who did not know. This could be related to the fact that patients with diabetes knowledge can adhere to medications, exercise, and self-care practices, including foot care, to control blood glucose [21,36], which helps them detect complications early and seek medical attention, thereby reducing hospitalization.

Patients in the age groups 50–59 and ≥60 were less likely to have diabetes-related admission, similar to findings from Australia [37]. However, this contradicts studies from Italy and England, where elderly patients were predictors of diabetes-related admission [11,16]. The discrepancy may be due to differences in duration of diabetes, presence of chronic complications, and adherence among the elderly. In our setup, the majority of elderly patients do not have chronic complications, have short-duration DM, and 67.4% of them have regular follow-up. This may lead to adequate glycemic control and decreased diabetes-related admission.

Older patients showed better adherence since these people might have more severe form of the disease compared to the other age groups [17].

In this study, patients with comorbidity were 3.27 times more likely to have diabetes-related admission, similar to findings from Italy and Harer [11,26]. Patients with obesity were 3.9 times more likely to have diabetes-related admission, comparable to findings from Italy, where obesity was an independent predictor [11]. This could be related to the fact that patients with comorbidity and high BMI (obese) are more likely to seek medical attention and hospital visits than their counterparts [11,16].

Regarding predictors of treatment outcome on multivariable analysis, poor exercise, hypertension, unsatisfactory knowledge about DM, and being unmarried were predictors of poor treatment outcomes.

Patients who have hypertension are 3.17 times more likely to have poor treatment outcomes than patients who do not have hypertension. This is comparable with findings in Saudi Arabia, where hypertension was a predictor of mortality [24]. Hypertension is common among diabetes patients and a strong risk factor for ASCVD, heart failure, and microvascular complications. This is presumed to be of atherosclerotic origin and is the leading cause of morbidity and mortality in diabetes patients [38].

Patients who have poor exercise practice are 2.41 times more likely to have poor treatment outcomes than patients who have good exercise practice. The majority of patients enrolled in this study (57.5%) had poor exercise practice before hospital admission, which in turn may increase blood glucose, increasing the patient’s susceptibility to infection and ultimately leading to poor treatment outcomes. Regular physical activity reduces the risk of raised blood glucose and is an important contributor to overall energy balance, weight control, and obesity prevention [39].

Patients with unsatisfactory knowledge about DM were 3.5 times more likely to have poor treatment outcomes. This could be explained by the fact that patients with poor knowledge about diabetes are less compliant with their medication and self-care practices, which results in poor glycemic control and leads to poor treatment outcomes.

Unmarried patients were 3.34 times more likely to have poor treatment outcomes. Being unmarried may increase social isolation, feelings of loneliness, lack of confidence in the community, health instability, and economic crisis. These factors may lead to poor medication adherence, delayed hospitalization, and ultimately poor treatment outcomes

### Strength and limitation of the study

The strength of this study is that it included the majority of wards in DURH. Also, the prospective nature of the study provides better data quality and comprehensive information. Though this study has the following limitations: Even if the sample size was higher compared to a previous prospective study done in Ethiopia, it was still small. The study looked at only a single facility and should be conducted as a multi-center study. We assessed self-care behavior, diabetes knowledge, and medication adherence based on participants’ self-report, which is subject to recall bias. However, this study gave useful insights into reasons for hospitalization, treatment outcomes, and predictors. It provides useful information for consultative, comparative, and future research purposes in the study center.

### Recommendations

Based on our findings, we recommend that healthcare professionals at DURH promote the treatment of patients with infections and comorbidities such as hypertension, cardiac conditions, and obesity. Healthcare professionals should also strengthen patient education on glycemic control and self-care practices, as the majority of patients show poor self-care and poor metabolic control. Health policy makers should consider developing certified diabetes educators who are trained to systematically support patients’ behavioral change efforts. Finally, a nationwide study should be conducted to identify the overall hospital admission patterns of diabetic patients.

## Conclusion

This study showed that the most common reasons for hospitalization of diabetic patients at DURH were hyperglycemic emergencies, infections, and CVDs. DKA was the most common cause of admission, followed by infectious diseases, with COVID-19 being the leading infection-related diagnosis. The majority of patients were discharged with improvement, while a significant number died in the hospital; mortality was higher among patients admitted due to infections. This level of mortality highlights the need for improved inpatient care and enhanced outpatient management for diabetic patients to reduce admissions due to preventable causes.

Above all, I would like to thank Jimma University and Dilla University.

## Abbreviations

BMI: Body Mass Index
CAD: Coronary Artery Disease
CHF: Congestive Heart Failure
COVID-19: coronavirus disease of 2019
CVDs: Cardiovascular Diseases
DFS: Diabetes Foot Syndrome
DKA: Diabetes Ketoacidosis
DM: Diabetes Mellitus
DURH: Dilla University Referral Hospital
HHS: Hyperglycemic Hyperosmolar State
HTN: Hypertension
IDF: International Diabetes Federation
JUSH: Jimma University Specialized Hospital
MMAPS: Modified Morisky Adherence Predictor Scale
MVC: Macro Vascular complication
RBS: Random Blood Sugar
SSNPR: Southern Nations, Nationalities and Peoples Region
T1DM: Type 1 Diabetes Mellitus
T2DM: Type 2 Diabetes Mellitus
UTI: urinary tract infection
WHO: World health organization
